# Sex Differences in the TyG Index and Cardiovascular Risk Factors in Metabolically Obese Normal Weight Phenotype

**DOI:** 10.1155/2022/1139045

**Published:** 2022-03-24

**Authors:** Xiaoyang Xu, Akshaya Srikanth Bhagavathula, Yong Zhang, Paul M. Ryan, Jamal Rahmani, Xiaoya Qi

**Affiliations:** ^1^Physical Examination Center, The Second Affiliated Hospital of Chongqing Medical University, Chongqing 400016, China; ^2^Department of Social and Clinical Pharmacy, Charles University, Hradec Kralove, Czech Republic; ^3^School of Public Health and Health Management, Chongqing Medical University, Chongqing, China; ^4^School of Medicine, University College Cork, Cork, Ireland; ^5^Cancer Research Center, Shahid Beheshti University of Medical Sciences, Tehran, Iran; ^6^Medical Data Science Academy, Chongqing Medical University, Chongqing, China

## Abstract

**Background:**

The triglyceride glucose (TyG) index is a novel surrogate marker of insulin resistance and increases cardiovascular disease risk. We sought to explore sex differences in the relationship between TyG and cardiovascular (CV) risk factors in metabolically obese normal weight (MONW) phenotype.

**Method:**

We analyzed data of 1208 healthy men and nonpregnant women enrolled in a population-based longitudinal study from January 2017–June 2020. MONW phenotype was defined by normal body mass index (BMI: 18–<25 kg/m^2^) with at least one of the following metabolic disorders (MONW phenotype): elevated blood pressure (BP), hypertriglyceridemia, hyperglycemia, and low HDL cholesterol. Multiple logistic regression analysis was performed to assess the association between elevated TyG index and the CV risk factors in women and men and was presented in odds ratio (OR) with 95% confidence interval (CI).

**Results:**

Of 1208 subjects, 350 (29%) were MONW phenotype (mean age (years): male: 43.5 ± 12.4 and female: 43.1 ± 12.7) and 858 were metabolically healthy normal weight (MHNW; *n* = 858 (71%)). MONW women had higher mean values of the TyG index (8.03 ± 5.07) than men (7.47 ± 4.68). Multivariate analysis revealed that the elevated TyG index is significantly associated with MONW phenotype in women (adjusted OR: 8.73, 95% CI: 5.62–13.57) and men (aOR: 5.90, 95% CI: 4.23–8.23). TyG was found to be an excellent predictor of MONW status in both women (receiver operating characteristic (ROC) area under the curve (AUC): 0.979, 95% CI: 0.969–0.988) and men (ROC-AUC: 0.968, 95% CI: 0.952–0.983).

**Conclusion:**

Our study revealed that the TyG index may represent a cost-effective and informative screening tool for the high-risk MONW phenotype.

## 1. Introduction

Individuals with normal weight but displaying obesity-related metabolic derangements are known as metabolically obese normal weight (MONW) [1, 2]. Despite displaying a normal body mass index (BMI: 18.5–25 kg/m^2^), these individuals exhibit derangements indicative of metabolic syndrome, such as hypertriglyceridemia (TG: ≥150 mg/dL), hyperglycemia (fasting blood glucose (FBS) ≥ 100 mg/dL), hypertension (≥130/85 mmHg), and also low high-density lipoprotein cholesterol (HDL: <40 mg/dL in men and <50 mg/dL in women) [2–5]. These characteristics confer a higher risk of cardiovascular disease (CVD) and type 2 diabetes (T2DM). However, not being overweight or obese, MONW subjects may escape detection and may not benefit from appropriate intervention programs. Therefore, early identification of these individuals could allow effective secondary prevention of CVD and T2DM in a group at high risk of developing cardiometabolic disease.

Recently, the triglyceride glucose (TyG) index, the product of TG and FBS, has emerged as a valuable marker to identify, among apparently healthy individuals, those at a higher risk of insulin resistance and CVD [6–9]. Several studies have examined the predictive value of the TyG index in terms of new-onset T2DM [10] and cardiovascular (CV) events [9] in seemingly healthy cohorts. Similarly, additional studies have investigated the usefulness of the TyG index in screening subjects at high risk of MONW phenotype [6, 11]. Nevertheless, the association of the TyG index with CV risk factors among MONW phenotype subjects remain unclear. Additionally, significant differences in the TyG index between were observed men and women in a recent study, with the metric most strongly associated with incident T2DM in women, regardless of their obesity status [12].

Therefore, we aimed to explore intersex variation in the relationship between the TyG index and CV risk factors in a cohort of MONW and metabolically healthy normal weight (MHNW) individuals enrolled in a Chinese population-based longitudinal study.

## 2. Methods

This retrospective cohort study evaluated the data of the general population undergoing routine physical and clinical examination in the health care centers of The Second Affiliated Hospital of Chongqing Medical University in Chongqing city, southwest China. The study was approved by The Second Affiliated Hospital of Chongqing Medical University Ethics Committee (no. 2020–252).

### 2.1. Study Population

Each year, around 100,000 residents of Chongqing city will take part in this routine examination in this center. For the present study, all generally healthy subjects with consecutive data from January 1, 2017 to June 2020 were screened and at last, 1840 (1088 men and 752 women) were considered. After excluding 569 people with overweight and obesity (BMI >25; 393 men and 176 women) or underweight (BMI <18.5; *n* = 63), a total of 1208 healthy individuals (657 men and 551 nonpregnant women) were included in the analysis.

### 2.2. Data Collection

The anthropometric data (weight, height, and waist and hip circumferences) and blood pressure were measured by trained staff following standard procedures. Bodyweight (kg) and height (cm) were measured in light clothing and without shoes with the use of a calibrated digital scale and stadiometer, respectively; body mass index (BMI) was calculated as weight in kilograms divided by height in meters squared (kg/m2). Waist and hip circumferences were measured in centimeters with a soft tape scale while participants were standing and wearing no heavy outer garments. Waist circumference (WC) was measured at the level of the umbilicus, and hip circumference was measured at the level of the bilateral greater trochanters. Waist-to-hip ratio (WHR) was computed as WC divided by hip circumference. Blood pressure and heart rate were measured by an automated procedure using the Omron digital monitor according to standard procedures.

Blood samples were taken by puncturing the cubital vein, under standardized conditions, between 7 : 30 and 12 : 00 a.m., with the participant having fasted at least 12 h beforehand. All blood samples were used to perform biochemical analyses by means of standard laboratory procedures. These analyses included plasma glucose (mg/dL), glycated hemoglobin (HbA1c, %), high-density lipoprotein cholesterol (HDL-C, mg/dL), low-density lipoprotein cholesterol (LDL-C, mg/dL), serum total cholesterol (TC, mg/dL), triglyceride (TG, mg/dL) levels, plasma uric acid (*μ*mol/L), aspartate aminotransferase (IU/L), and alanine aminotransferase (IU/L).

### 2.3. Operational Definitions

Subjects were allotted into the MONW or MHNW group based on their cardiometabolic profile. MONW phenotype was defined by BMI ≥18.5 to <25 kg/m^2^ and presence of at least one of the following CV risk factors such as TG levels ≥150 mg/dL, HDL <40 mg/dL in men and <50 mg/dL in women, values of systolic blood pressure (SBP) ≥130 mmHg or diastolic blood pressure (DBP) ≥85 mmHg, and FBS ≥100 mg/dL [13]. The TyG index was calculated as follows [14]:

TyG index = Ln [fasting TG (mg/dl) × FBG (mg/dl)]/2.

The optimal TyG cut-off value used for the elevated TyG index was 5.76 for men and 6.82 for women [15].

### 2.4. Data Analysis

Data were analyzed using SPSS for Windows version 24.0 (SPSS Inc., Chicago, IL). Categorical variables are expressed as proportions, and continuous variables are shown as mean ± standard deviation (SD). For glucose and triglycerides, coefficient of variation (SD/mean×100%) was calculated to compare the variability. Differences between men and women were examined using Student's *t*-test or Mann–Whitney *U* test for continuous variables and chi-square test for categorical variables. The correlation between the TyG index and CV risk factors such as elevated BP (SBP ≥130 mmHg and DBP ≥85 mmHg), FBS, HDL-C, and TG levels was estimated using the Spearman correlation test. The association between the elevated TyG index and MONW using multiple logistic regression in women and men separately. The logistic regression models were adjusted for age, BMI, and waist circumference (WC) to predict the level of association between the elevated TyG index and CV risk factors. Odds ratios (OR) and 95% confidence intervals were calculated. The above analysis was repeated separately for men and women in the entire cohort. Finally, receiver operating characteristics (ROC) curves were generated, and area under curve (AUC) analyses were performed to assess the sensitivity and specificity of the TyG index in detecting the MONW phenotype by sex. The ROC curve evaluates the relationship between sensitivity and specificity for every possible cut-off in the curve. In the ROC curve, the *y*-axis is sensitivity (sensitivity = true positive/true positive + false negative) and *x*-axis is 1-specificity. A *P* value of <0.05 was considered statistically significant.

## 3. Results

### 3.1. Characteristics of the Study Population

Among 1208 subjects, 350 (29%) individuals exhibit the MONW phenotype (53.7% women) and 71% are MHNW (57.7% men). Anthropometric and biochemical characteristics of male and female subjects of MONW and MHNW groups are shown in Table 1. Coefficient of variation showed that triglycerides are relatively more variable than glucose. In the MONW group, women exhibited lower WC (65.35 vs. 76.70 cm; *P* < 0.001) and higher total cholesterol levels (TC; 204.46 vs. 200.75 mg/dL; *P* = 0.201), FBS (99.40 vs. 94.94 mg/dL; *P* = 0.003), HDL-C (54.54 vs. 47.86 mg/dL; *P* = 0.102), TG (250.69 vs. 247.82; *P* = 0.564), and TyG index (8.03 vs. 7.47; *P* = 0.140) than men, although only WC and FBS were statistically significant in their intersex discordance. Conversely, within the MHNW group, women had statistically higher FBS (94.03 vs. 89.07 mg/dL; *P* < 0.001), TC (185.53 vs. 172.14 mg/dL; *P* < 0.001), HDL-C (54.84 vs. 42.37; *P* < 0.001), TG (101.12 vs. 95.87 mg/dL; *P* = 0.021), and TyG index (3.07 vs. 2.88; *P* = 0.022) than men. Overall, the frequency of hypertension was 10.9% and 8%, hypertriglyceridemia was 53.7% and 46.3%, hyperglycemia was 16.6% and 10.3%, and low HDL-C was 5% and 3.8% for women and men, respectively.

### 3.2. TyG Index in MONW Phenotype

**Figure 1:**
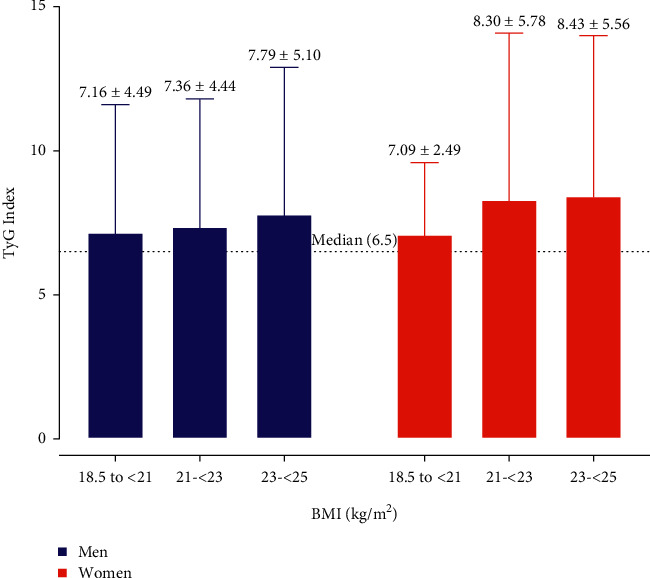
Sex differences in the TyG index among metabolically obese normal weight subjects.

The mean TyG index in the MONW group was 7.77 ± 4.89 (SD), with women displaying a statistically insignificant higher TyG index (8.03 ± 5.07) than men (7.47 ± 4.68). We performed subgroup analysis by stratifying the BMI into three groups: 18.5 to <21 kg/m^2^, 21 to <23 kg/m^2^, and 23 to <25 kg/m^2^, and the variations in the TyG index among women and men are shown in Figure 1. This comparison showed no significant difference in TyG index in both the groups (*P* = 0.289), with a higher mean TyG index in women with BMI: 23 to <25 kg/m^2^ (8.43 ± 5.56) and 21 to <23 kg/m^2^ (8.30 ± 5.78) than their BMI-matched male counterparts (7.79 ± 5.10 and 7.36 ± 4.44).

### 3.3. Correlation of the TyG Index with CV Risk Factors

The Spearman correlation test showed a significant positive correlation of the TyG index with CVD risk factors such as hyperglycemia (female: *r*^2^ = 0.358, *P* < 0.001; male: *r*^2^ = 0.357, *P* < 0.001), hypertriglyceridemia (female: *r*^2^ = 0.822, *P* < 0.001; male: *r*^2^ = 0.787, *P* < 0.001), and low HDL-C (female: *r*^2^ = 0.142, *P* = 0.003; male: *r*^2^ = 0.115, *P* = 0.018) in both men and women (Table 2). However, no association between TyG and hypertension was identified for either sex (female: *r*^2^ = −0.057, *P* = 0.179; male: *r*^2^ = −0.034, *P* = 0.390).

### 3.4. Association between the TyG Index and MONW Phenotype

The unadjusted logistic regression analysis showed a significant association between elevated TyG and MONW phenotype (OR: 4.74, 95% CI: 5.24–8.68) and higher odds in women (OR: 8.25, 95% CI: 5.44–12.51) than men (OR: 5.79, 95% CI: 4.22–7.95). After logistic regression analysis adjusted by age, BMI, and WC, the elevated TyG index remained significantly associated in women (aOR: 8.73, 95% CI: 5.62–13.57) and men (aOR: 5.90, 4.23–8.23).

### 3.5. Association between the TyG Index and CV Risk Factors

Multiple logistic regression analysis of the association between the TyG index and CV risk factors in the women and men with MONW phenotype is shown in Table 3. The unadjusted analysis indicated a significant association between elevated TyG index with low HDL-C levels, hyperglycemia, and hypertriglyceridemia in women and men. In the adjusted analysis, the elevated TyG index remained significantly associated with higher odds of hypertriglyceridemia (aOR: 113.0, 95% CI: 38.82–328.96) in women than men (aOR: 101.6, 95% CI: 35.93–287.73). In men, low HDL-C (aOR: 16.9, 95% CI: 0.25–229.6) and hyperglycemia (aOR: 7.5, 95% CI: 4.16–13.57) were significantly associated with the elevated TyG index in MONW phenotype but not with hypertension (Table 3).

### 3.6. Accuracy of the TyG Index for Identifying MONW Phenotype by Sex

**Figure 2:**
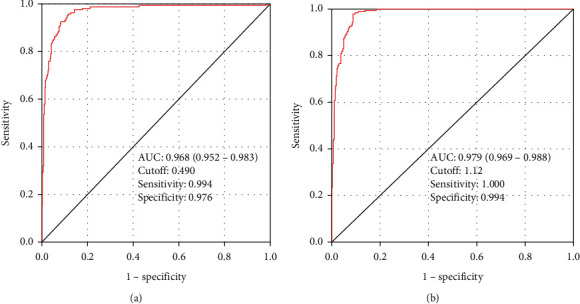
Receiver operating characteristics analysis of the TyG index for the identification of metabolically obese normal weight phenotype in (a) men and (b) women.

We further assessed the diagnostic accuracy of the TyG index stratified by sex. As shown in Figure 2, the strength of the TyG index in the detection of MONW phenotype was more significant in women than in men. The AUC was 0.979 (95% CI: 0.969–0.988; sensitivity: 1.000; specificity: 0.994) in women and 0.968 (95% CI: 0.952–0.983; sensitivity: 0.994; specificity: 0.976) men subjects, respectively.

## 4. Discussion

Those displaying the MONW phenotype represent a cohort of individuals who may benefit greatly from intervention in the form of secondary prevention of CVD and T2DM. However, such populations are challenging to detect in clinical practice due to their inherent lack of overt obesity, which is at the core of metabolic syndrome and an obligatory factor in its diagnosis, as per the International Diabetes Foundation [16]. Therefore, the development of suitably sensitive, specific, and cost-effective screening tests for such individuals is indeed desirable. In the present study, we examined the utility of the TyG index as such a marker in 1208 normal-weight Chinese men and women, with and without metabolic derangements. This revealed that nearly one-third of an otherwise seemingly healthy population may be classed as MONW and therefore, deemed at-risk of CVD or T2DM development. Additionally, the TyG index was found to be highly sensitive and specific for the detection of the MONW phenotype.

A range of observational studies has established the MONW phenotype as a risk factor in the development of cardiometabolic disease and early mortality [17]. Indeed, in the southwest Seoul study of 2317 elderly Korean individuals, all-cause and CVD-related mortalities at 10-year follow-up were both significantly greater in metabolically healthy obese individuals when compared to their age-matched MONW counterparts [18]. However, long-term follow-up data on the clinical outcomes, in terms of events and mortality, are not yet available for the present cohort; meaning that accurate risk stratification with the TyG is limited at present. Nevertheless, the use of the marker in identifying individuals with the MONW phenotype will support such research in the area.

There is evidence to suggest that improvements in dietary quality in MONW individuals confers significant risk reduction in terms of all-cause and CVD mortality [19]. This is would make the TyG index a useful tool to enable primary care practitioners to rapidly identify a population which would benefit clinically from dietary and lifestyle intervention or education. However, despite the utility of the TyG index in predicting MONW, it was not found to be a useful predictor of hypertension in the present cohort. This is in keeping with other reports [20] and indicates that a comprehensive clinical examination and ambulatory blood pressure monitoring, where available, remain indispensable for complete risk stratification in such populations.

It has previously been noted that, although MONW individuals maintain a normal BMI, their fat distribution is less favorable in that it is primarily truncal or visceral adipose tissue [21]. Intriguingly, MONW status was not associated with an increased WC compared to MHNW participants in the present cohort. This suggests that WC alone may not suffice as an adjunct screening tool to identify MONW individuals in such a population and therefore, biochemical investigations such as the TyG index may instead be desirable.

A previous investigation involving 7602 participants of the Third National Health and Nutrition Examination Survey found that, even within the normal BMI range, the prevalence of metabolic derangements increased substantially with BMI [20]. However, this effect was most amplified in the female subcohort. Similarly, there appears to be significant intersex variation in the TyG index level and its association with HDL-C in MONW individuals, which was found to be substantially stronger in males, in the present cohort. As a result, the TyG index normal range must be established independently for both sexes and such discordance needs to be taken into consideration if the marker proves useful in clinical risk stratification.

To our knowledge, the present study is the first report assessing the applicability and validity of the TyG index for detection of the MONW phenotype and associated CVD risk factors in a population of normal-weight Chinese participants. The main strength of this study is to assess the sex differences in the TyG index and low HDL-C. Additionally, with 1208 participants, this cohort is substantially larger than the previously available reports of relevance. The main limitation of this study is that the population come from one center which may introduce selective bias. Besides, another one is that correlation of the TyG index with clinically relevant outcomes, such as myocardial infarction, cerebrovascular event and T2DM development, was not available for the present cohort. Collection of data which will allow validation of this marker as a predictor of outcomes and events remains a priority, as this would allow for more accurate risk stratification through the TyG index.

## 5. Conclusion

In the present cohort of 1208 seemingly healthy, normal-weight Chinese participants, some form of intervenable metabolic derangement was identified in nearly one-third of individuals. Furthermore, the TyG index was found to be highly sensitive and specific for the MONW phenotype amongst this cohort. Therefore, the TyG index may be a useful screening tool in identifying normal-weight individuals who may be at elevated risk of cardiometabolic disease in a primary care setting. Future research is required on this topic and also to examine the utility of this potential marker in predicting the risk of clinically relevant outcomes and events.

## Figures and Tables

**Table 1:** Characteristics of the study population stratified by sex (*n* = 1208).

	MONW (*N* = 350, 29%)			MHNW (*N* = 858, 71%)		
	Women	Men	*P* value	Women	Men	*P* value
Total (%)	188 (53.7)	162 (46.3)	—	363 (42.3)	495 (57.7)	—
Age	43.49 ± 12.48	43.16 ± 12.75	0.630	46.18 ± 14.20	45.49 ± 12.70	0.868
BMI, kg/m^2^	22.14 ± 1.61	22.32 ± 1.73	0.271	22.19 ± 1.68	22.21 ± 1.71	0.834
Waist circumference, cm	65.35 ± 22.16	76.7 ± 17.65	**<0.001** ^∗^	68.40 ± 22.17	79.52 ± 18.89	**<0.001** ^∗^
SBP, mmHg	118.98 ± 13.94	118.24 ± 14.96	0.371	119.49 ± 14.06	118.25 ± 13.96	0.188
DBP, mmHg	72.13 ± 8.43	72.18 ± 9.57	0.674	72.61 ± 9.16	71.57 ± 8.93	0.102
Total cholesterol, mg/dL	204.46 ± 37.68	200.75 ± 37.33	0.201	185.53 ± 33.41	172.14 ± 38.02	**<0.001** ^∗^
HDL cholesterol, mg/dL	54.54 ± 28.62	47.86 ± 33.49	0.102	54.84 ± 30.65	42.37 ± 33.50	**<0.001** ^∗^
TyG index	8.03 ± 5.07	7.47 ± 4.68	0.140	3.07 ± 2.88	2.88 ± 1.20	**0.022** ^∗^
Fasting glucose, mg/dL	99.40 ± 20.53 (CoeV = 22.7%)	94.94 ± 19.92 (CoeV = 20.2%)	**0.003** ^∗^	94.03 ± 16.70 (CoeV = 17.8%)	89.07 ± 18.44 (CoeV = 20.7%)	**<0.001** ^∗^
Triglycerides, mg/dL	250.69 ± 140.08 (CoeV = 55.9%)	247.82 ± 146.08 (CoeV = 58.9%)	0.564	101.12 ± 27.79 (CoeV = 27.5%)	95.87 ± 30.85 (CoeV = 32.2%)	**0.021** ^∗^

^∗^represents statistical significance (P value < 0.05)

**Table 2:** Sex differences in the Spearman correlation between TyG index and cardiovascular risk factors.

Parameters	Women		Men	
	*r* ^2^	*P* value	*r* ^2^	*P* value
Hypertension (≥130/≥85 mmHg)	−0.057	0.179	−0.034	0.390
Hyperglycemia (≥100 mg/dL)	0.358	<0.001	0.357	<0.001
Low HDL cholesterol (<40 men/<50 women mg/dL)	0.142	0.003	0.115	0.018
Hypertriglyceridemia (≥150 mg/dL)	0.822	<0.001	0.787	<0.001

**Table 3:** Logistic regression analysis evaluating the sex differences in the association between the TyG index and cardiovascular risk factors in the MONW phenotype.

Parameters	Unadjusted OR (95% CI)				Adjusted OR (95% CI)			
	Women	*P* value	Men	*P* value	Women	*P* value	Men	*P* value
Hypertension (≥130/≥85 mmHg)	1.06 (0.64–1.75)	0.819	0.63 (0.32–1.24)	0.185	1.02 (0.58–1.81)	0.922	0.78 (0.38–1.61)	0.511
Hyperglycemia (≥100 mg/dL)	**4.77 (2.99–7.61)**	**<0.001** ^∗^	**5.00 (2.92–8.55)**	**<0.001** ^∗^	**4.79 (2.95–7.77)**	**<0.001**	**7.51 (4.16–13.57)**	**<0.001** ^∗^
Low HDL cholesterol (<40/<50 mg/dL)	**2.58 (1.04–6.38)**	**0.040** ^∗^	**5.52 (2.23–13.64)**	**<0.001** ^∗^	**2.75 (1.10–6.91)**	**0.031**	**16.96 (1.25–229.6)**	**0.033** ^∗^
Hypertriglyceridemia (≥150 mg/dL)	**105.33 (37.59–295.10)**	**<0.001** ^∗^	**98.45 (35.29–274.65)**	**<0.001** ^∗^	**113.01 (38.82–328.96)**	**<0.001**	**101.68 (35.93–287.73)**	**<0.001** ^∗^

^∗^represents statistical significance (P value < 0.05)

## Data Availability

Datasets are available from the corresponding author on reasonable request.

## Additional Points

TyG is an excellent predictor of MONW status. TyG puts both participants at higher risk of low HDL-C (men and women). The TyG index predicted a higher CV risk in the Chinese population with the MONW phenotype.
